# Potential of Zerumbone as an Anti-Cancer Agent

**DOI:** 10.3390/molecules24040734

**Published:** 2019-02-18

**Authors:** Sosmitha Girisa, Bano Shabnam, Javadi Monisha, Lu Fan, Clarissa Esmeralda Halim, Frank Arfuso, Kwang Seok Ahn, Gautam Sethi, Ajaikumar B. Kunnumakkara

**Affiliations:** 1 Cancer Biology Laboratory, DBT-AIST International Laboratory for Advanced Biomedicine (DAILAB), Department of Biosciences & Bioengineering, Indian Institute of Technology, Guwahati, Assam 781039, India; sosmi176106101@iitg.ac.in (S.G.); bano176106104@iitg.ac.in (B.S.); j.monisha@iitg.ernet.in (J.M.); 2 Department of Pharmacology, Yong Loo Lin School of Medicine, National University of Singapore, Singapore 117600, Singapore; phcfanl@nus.edu.sg (L.F.); phsceh@nus.edu.sg (C.E.H.); 3 Stem Cell and Cancer Biology Laboratory, School of Pharmacy and Biomedical Sciences, Curtin Health Innovation Research Institute, Curtin University, Perth, WA 6102, Australia; frank.arfuso@curtin.edu.au; 4 College of Korean Medicine, Kyung Hee University, 24 Kyungheedae-ro, Dongdaemun-gu, Seoul 02447, Korea

**Keywords:** zerumbone, cancer, apoptosis, NF-κB, IL-6/JAK2/STAT3, Akt, FOXO1

## Abstract

Cancer is still a major risk factor to public health globally, causing approximately 9.8 million deaths worldwide in 2018. Despite advances in conventional treatment modalities for cancer treatment, there are still few effective therapies available due to the lack of selectivity, adverse side effects, non-specific toxicities, and tumour recurrence. Therefore, there is an immediate need for essential alternative therapeutics, which can prove to be beneficial and safe against cancer. Various phytochemicals from natural sources have been found to exhibit beneficial medicinal properties against various human diseases. Zerumbone is one such compound isolated from *Zingiber zerumbet* Smith that possesses diverse pharmacological properties including those of antioxidant, antibacterial, antipyretic, anti-inflammatory, immunomodulatory, as well as anti-neoplastic. Zerumbone has shown its anti-cancer effects by causing significant suppression of proliferation, survival, angiogenesis, invasion, and metastasis through the molecular modulation of different pathways such as NF-κB, Akt, and IL-6/JAK2/STAT3 (interleukin-6/janus kinase-2/signal transducer and activator of transcription 3) and their downstream target proteins. The current review briefly summarizes the modes of action and therapeutic potential of zerumbone against various cancers.

## 1. Introduction

**Figure 1:**
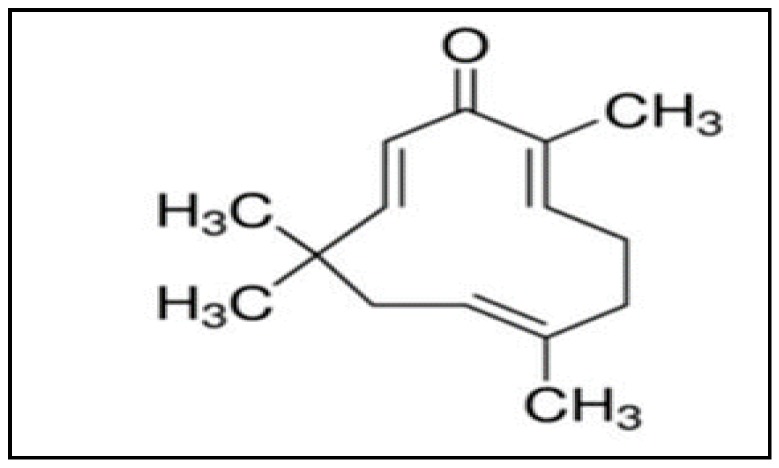
Structure of zerumbone [38].

Cancer is a serious health problem globally and is the second leading cause of death [1,2,3,4,5]. The risk factors for cancer mortality may be due to behavioural and environmental factors such diet, exposure to air pollution, use of addictive substances, lack of physical activity, etc. [6]. The recent targeted therapies for cancer are associated with non-specific toxicities, exhibit limited efficacy, and are expensive to use [7]. Also, one of the major drawbacks of anti-cancer drugs has been the lack of selectivity, and thus they can exert adverse effects on the healthy tissues and organs [8]. Furthermore, conventional chemotherapeutics are also associated with the development of chemoresistance [9,10,11,12]. Overall, most cancer treatment strategies are associated with side effects, lack of selectivity, toxicity, and development of chemoresistance, leading to high mortality and poor prognosis [7,8,9,10,11,12,13]. Herbs, vegetables, fruits, spices, cereals, pulses, and nuts have been shown to produce a large number of phytochemicals that exert chemopreventive and therapeutic properties against various human ailments [14,15,16,17,18,19,20,21,22,23,24,25,26,27,28,29]. Since ancient times, plants and plant-based compounds have been extensively used in tribal and folklore medicine due to their beneficial pharmacological properties and fewer side effects [20,30,31,32]. One such plant-derived compound is zerumbone (Figure 1), extracted from the traditional plant *Zingiber zerumbet* Smith, which is also known as bitter ginger, shampoo ginger, and pinecone ginger [33,34,35,36,37,38]. 

Traditionally, the rhizomes of *Z. zerumbet* are widely used as a food flavouring agent and appetizer in various cuisines; it is also used in herbal folk medicine and pain relief [37,38,39,40]. Zerumbone is known for its biomedical properties such as being antioxidant, antibacterial, antipyretic, antinociceptive, anti-hypersensitive, anti-inflammatory, as well as possessing hepatoprotective, and immunomodulatory activities [16,17,41,42,43,44]. Besides the aforementioned properties, zerumbone can also function as an antitumor drug exhibiting its diverse effects on proliferation, angiogenesis, and apoptosis against various cancer cell lines, which has drawn the attention of many researchers [45,46,47]. Zerumbone (2,6,9,9-tetramethyl-(2*E*,6*E*,10*E*)-cycloundeca-2,6,10-trien-1-one) is a crystalline sesquiterpene compound containing an 11-membered ring with three double bonds at C6, C2, and C10, forming part of a cross-conjugated ketone system that participates in its biological activity [48,49,50,51]. A number of prior studies have elaborated upon the potential anti-allergic, immunomodulatory, and anti-cancer properties of zerumbone. The present review summarizes the anti-cancer properties of zerumbone along with its mode of actions and molecular targets in different cancer types.

## 2. Molecular Targets of Zerumbone against Cancer

It is now well established that cancer arises as a result of dysregulation of multiple cellular pathways. Therefore, multi-targeted compounds have high potential in the prevention and treatment of cancer [2,5,18,26,52,53,54,55,56,57,58]. Increasing lines of evidence suggest that zerumbone can act on multiple pathways and can suppress the growth of cancer cells. With regard to this, in melanoma zerumbone treatment downregulated Bcl-2 (B-cell lymphoma 2), upregulated Bax (BCL2 associated X protein), cytochrome c gene-related proteins, and activated caspase-3 [59]. In addition, zerumbone decreased cell viability through the downregulation of cyclin B1, cyclin-dependent kinase (CDK)-1, Cdc25C, and Cdc25B, and induced apoptosis by significantly activating Bax and Bak proteins in breast cancer [60]. Moreover, zerumbone downregulated CXCR4 expression on human-epidermal growth factor receptor-2 (HER2)-overexpressing breast cancer cells by abrogating NF-κB activation which correlated with the arrest of invasion and metastasis induced by CXCL12 expression [61]. In addition, zerumbone could also activate caspase-3 and poly (ADP-ribose) polymerase (PARP) production and reduced Akt phosphorylation, which increased cell death induction in glioblastoma [62]. Further, the treatment of zerumbone on mouse epidermal JB6 cells can induce an increase in NF-E2-related factor 2(Nrf2) nuclear translocation complemented by the upregulation of heme oxygenase-1 (HO-1), causing Nrf2 to bind directly to the antioxidant response element, and thus exert chemopreventive effects against mouse skin carcinogenesis [63]. In addition, zerumbone inhibited breast cancer and multiple myeloma-induced osteoclast formation through suppression of activated IkappaBalpha (IκBα) kinase (IKK), IκBα phosphorylation, and IκBα degradation, which led to the abrogation of receptor activator of nuclear factor kappa-Β ligand (RANKL)-induced NF-κB activation, which serves as a major mediator for bone resorption [64]. Zerumbone could also induce apoptosis in leukemic cells through the initiation of the cleavage of Bid, Bax, and Mcl-1 proteins, loss of the mitochondrial transmembrane potential, and activation of caspase-3 and -9, leading to degradation of PARP [65]. 

Natural products possess several properties, which enable them to fight against various cancer cell types [34,66]. Zerumbone possesses antiproliferative properties against various cancers such as brain, breast, cervical, colon, liver, lung, pancreas, and skin cancer [3,67]. The reported anti-tumour activities of zerumbone against different malignancies are briefly summarized below. 

### 2.1. Zerumbone and Brain Tumor

Brain tumors are the leading cause of cancer-related death in children [68,69]. The incidence of primary malignant brain tumours is increasing, of which 80% consist of high-grade malignant tumors such as glioblastoma multiforme (GBM) [70]. Prior studies have reported that treatment with zerumbone suppressed FOXO1 and Akt phosphorylation due to inactivation of IKKα while activating caspase-3 protein and PARP, which resulted in decreased cell viability, and induction of apoptosis in GBM cells [62].

### 2.2. Zerumbone and Breast Cancer

Breast cancer is the most common and highly complex cancer associated with poorest clinical outcome in women worldwide [71,72,73,74,75,76]. Among the various subtypes, triple-negative breast cancer (TNBC) is the most aggressive form and, occurs in young age pre-menopausal women [77,78]. Triple-negative breast cancer fails to express the estrogen receptor (ER), human-epidermal growth factor receptor-2 (HER2) proteins, and progesterone receptor (PR) [77]. Zerumbone was found to increase the induction of presenilin-1 protein and transcriptional activation of Notch, leading to cleavage of Notch2. In addition, there was a reduction in cleaved Notch1 and Notch4 proteins, which resulted in increased apoptosis and suppression of cellular migration [79]. Zerumbone also decreased the levels of IL-8 and MMP-3 expression through the downregulation of NF-κB activity, which led to reduction in IL-1β-induced cell migration and invasion in TNBC [72,80]. Zerumbone induced G(2)/M cell arrest and Bax/Bak-mediated apoptosis [60]. Zerumbone also inhibited invasion and metastasis in breast cancer by downregulating the expression of CXCR4 [61]. Further, zerumbone suppressed TGF-β1-induced FN, MMP-2, and MMP-9 expression through the decreased phosphorylation of TGF-β1-induced Smad3, and also inhibited Ki67 expression [77].

Further, in mouse xenografts, zerumbone treatment was noted to reduce the tumor growth [60]. In addition, zerumbone decreased breast cancer-associated osteolysis in athymic nude mice with MDA-MB-231 breast cancer [64]. The mice treated with 20 mg/kg of zerumbone were observed to develop significantly smaller tumors than the control group treated with vehicle. Thus, zerumbone mitigated the tumorigenicity by reducing tumor volume and weight, and metastasis in xenograft models of TNBC [77].

### 2.3. Zerumbone and Colorectal Cancer

Colorectal cancer (CRC) is the third-leading cause of cancer-related deaths after lung and breast cancer [81,82]. Zerumbone inhibited the proliferation of colon cancer cells and thereby induced apoptosis, which might be due to mitochondria transmembrane dysfunction, translocation of phosphatidylserine, and chromatin condensation [83]. Zerumbone downregulated the expression of FLICE-like inhibitory protein (cFLIP); and in the presence of both Bax and p21, stimulated tumor necrosis factor-related apoptosis-inducing ligand (TRAIL) death receptor (DR)4 and DR5, which intensified TRAIL-induced apoptosis in human HCT116 colon cancer cells, thus resulting in the enhanced anticancer effects of TRAIL [84]. Zerumbone pre-treatment inhibited the expression of radiation-induced DNA repair proteins ataxia-telangiectasia mutated (ATM) and DNA-PKcs through glutathione (GSH) depletion, leading to cell cycle arrest (G2/M) and increase in apoptosis, thus enhancing radiosentitivity of CRC cells [82]. Zerumbone induced IL-1β pathways through the expression of interleukin (IL)-1α, IL-1β, IL-6, and tumor necrosis factor (TNF)-α, which might be associated with the activation of c-Jun N-terminal kinase and extracellular signal-regulated protein kinase [85]. Further, zerumbone treatment on HCT-116 and SW48 cells showed its anti-metastatic potential through the inhibition of Fak/PI3k/NF-κB-uPA pathway [86].

Oral administration of zerumbone at 100, 250, and 500 ppm for the duration of 17 weeks to the mice significantly inhibited the multiplicity and inflammation in colonic adenocarcinomas through the repression of NF-κB and heme oxygenase (HO)-1, which resulted in suppression of the proliferation and induction of apoptosis [87].

### 2.4. Zerumbone and Cholangiocarcinoma 

Cholangiocarcinoma (CCA) is an aggressive and deadly malignant tumor arising in the bile duct epithelial cells [88,89,90]. It is characterized by a high degree of genetic instability contributing to a heterogeneous malignant phenotype, and this variation is related to the distribution of different risk factors [91,92]. Surgical resection continues to be the best therapeutic approach for patients with CCA. Nevertheless, it is often associated with poor prognosis, and due to lack of early diagnostic evidence, most patients are not suitable for surgery [93]. The inhibition of CDK-2, CDK-5 or EGFR could result in reduction in CCA cell proliferation, and previous reports have revealed the overexpression of CDK-2, CDK-5 or EGFR in CCA. Molecular docking studies have demonstrated that derivatives of zerumbone with the presence of amine, epoxyamine, hydroxylamine, and nitrile groups can exhibit potent anti-cancer activities against CCA cells by interacting with the molecular target EGFR, but not CDK-2, CDK-5, and GSK-3. The derivative with the presence of an amine group has shown higher potency against proliferation of cancer cells through the inhibition of EGFR [49].

### 2.5. Zerumbone and Gastric Cancer

Gastric cancer is the fourth most common malignant tumor and second main cause of cancer deaths in both sexes worldwide [13,94,95,96,97,98]. Zerumbone could induce apoptosis in gastric cancer cells by blocking the activity of cyclophilin A and subsequently promoting mitochondrial pathway-mediated apoptosis. The mitochondrial pathway caused cyt C release from the mitochondria into the cytoplasm, leading to caspase-3 activation, which acted as an effector molecule for caspase-dependent apoptosis [13]. In addition, zerumbone caused inhibition of cell proliferation and tube formation area of human umbilical vein endothelial cells through the reduction in expression of vascular endothelial growth factor (VEGF) and NF-κB activities, and thereby inhibiting angiogenesis [99].

### 2.6. Zerumbone and Leukemia

Leukemia is the most common cancer that occurs in the blood forming tissues [5,100]. Zerumbone was reported to reduce the proliferation of leukemic cells by inducing G2/M cell cycle arrest through suppression of cyclin B1/cdk 1 protein along with the phosphorylation of ATM/Chk1/Chk2, followed by apoptosis via initiation of Fas (CD95)/Fas Ligand (CD95L) expression associated with caspase-8 activation [65,101]. Analogous to the abovementioned anti-proliferative effects, the use of a zerumbone-loaded nanostructured lipid carrier (NLC) arrested Jurkat cells in the G2/M phase via inactivation of cyclin B1 protein and activation of intrinsic apoptotic proteins (i.e., caspase-3 and caspase-9), and release of cytochrome c with subsequent cleavage of PARP [102,103]. Zerumbone-NLC also induced activation of the mitochondrial-dependent apoptotic pathway in murine leukemic cells [104]. Zerumbone inhibited K562 chronic myelogenous leukemic cell proliferation and colony formation, with induction of DNA damage and mitochondria mediated apoptosis through the activation of pro-caspase-3, -9, and PARP cleavage, with an increase in free intracellular Ca^2+^, ROS, and soluble histone-H2AX (H2A histone family member X) upregulation [105]. Additionally, zerumbone exerted a cytotoxic effect against CEM-ss leukemic cells by producing characteristics of apoptosis such as membrane blebbing, holes, and cytoplasmic extrusions [100].

### 2.7. Zerumbone and Liver Cancer

Hepatocellular carcinoma (HCC) is one of the most prevalent and highly aggressive liver malignancy [106,107,108,109,110,111,112,113,114]. Zerumbone has been shown to cause the inhibition of HCC cells proliferation by G2-M cell cycle arrest through the inhibition of PI3K/AKT/mTOR and signal transducer and activator of transcription 3 (STAT3) signalling pathways leading to the induction of apoptosis [115]. Zerumbone also inhibited the proliferation and migration of a liver cancer HepG2 cell line by significantly decreasing the expression of the MMP-9, VEGF, and VEGF receptor proteins in a dose-dependent manner [116]. Further, zerumbone exhibited also induced apoptosis via upregulation of Bax and suppression of Bcl-2 protein expression on HepG2 cells [117]. In line with the above, zerumbone treatment in DEN/AAF (diethylnitrosamine/2-acetylaminofluorene) rat livers induced mitochondria-regulated apoptosis by increasing Bax and decreasing Bcl-2 protein expression, along with a reduction of oxidative stress and an inhibition of proliferation, and thereby minimizing DEN/AAF-induced carcinogenesis [67]. Zerumbone can activate the Nrf2/ARE-dependent detoxification pathway induced by nuclear localization of Nrf2, which can bind to the antioxidant response element of the phase II enzyme genes, thereby suggesting an antioxidant role of zerumbone in neutralizing lipid peroxidation in liver cells to put a stop to cancer [48]. A soluble complex of zerumbone with hydroxypropyl-β-cyclodextrin (HP-β-CD) induced apoptosis and G2/M arrest along with the release of cytochrome c and loss of mitochondrial membrane potential, with a significant increase in caspase 3/7, caspase 9, and caspase 8, and with the depletion of BID that is cleaved by caspase 8 [118].

### 2.8. Zerumbone and Lung Cancer

Lung cancer is one of the most common malignancies and leading cause of cancer-related death worldwide [119,120,121,122]. About 80% of diagnosed lung cancer is non-small cell lung cancer (NSCLC), which ranks top in both incidence and mortality [123,124]. Zerumbone induces apoptosis through loss of mitochondrial membrane potential, release of cytochrome c, caspase-9 and -3 activation, increased expression of p53 and Bax, and increased ROS production, and zerumbone also sensitized NSCLC cells to cisplatin [125]. 

Further, dietary administration of zerumbone at 250 and 500 ppm to the mice for 21 weeks was found to significantly inhibit the multiplicity of lung cancer in a dose-dependent manner. The suppression of lung carcinogenesis was caused through the reduction of growth, decreased inflammation, and decreased expression of NF-κB and HO-1, thereby causing apoptosis [87].

### 2.9. Zerumbone and Oral Cancer

Oral cancer is a serious cancer in developing countries and many parts of the globe, with varying incidence rate according to geographic regions [126,127]. It affects approximately 274,000 people worldwide annually and the pattern of use of tobacco products usually marks the frequency of occurrence [128]. Zerumbone treatment inhibited cell proliferation, migration, and invasion in oral squamous cell carcinoma by suppressing the expression of CXCR4, RhoA proteins, and PI3K-mTOR signalling pathway, causing G2/M cell cycle arrest followed by apoptotic activity. The inhibition of the PI3K-mTOR signalling pathway was associated with the suppression of Akt and S6 proteins [129]. 

### 2.10. Zerumbone and Cervical and Ovarian Cancer

Cervical cancer ranks as the second most common cause of cancer death among women [130]. Zerumbone along with cisplatin treatment can stimulate apoptosis by arresting cells at the G2/M phase and decreasing the levels of IL-6 in HeLa and Caov-3 cells [131]. Zerumbone also downregulated the expression of proliferating cellular nuclear antigen, thus revealing its antitumor effect on human cervical cancer cells [132]. Similarly, zerumbone can induce apoptosis by causing regression of cervical intraepithelial neoplasia (CIN) tissues in female Balb/c mice treated with diethylstilbestrol, which showed an effect resembling that of cisplatin, through the modulation of Bax and Bcl-2 genes [130]. Zerumbone showed its cytotoxic property on HeLa cells by increasing cellular caspase-3 levels and producing distinct morphological features of apoptotic death such as nuclear and chromatin condensation, cell shrinkage, membrane blebbing, abnormalities of mitochondrial cristae, multinucleation, and formation of apoptotic bodies [133].

Also, the combination of zerumbone with cisplatin has been shown to cause regression of (CIN) via modulation of the IL-6 level in the serum of female Balb/c mice. Four groups of mice were taken for the treatment along with a group with no treatment from 52 days of age to 60 days of age with four dosages on alternate days. Group 1 mice were treated with 0.9% normal saline for the positive control while group 2 and group 3 mice were administered with 8 mg/kg and 16 mg/kg of zerumbone, respectively. The mice representing group 4 was treated with 10 mg/kg while group 5 was given no treatment to act as a negative control. Both the compounds, at their mentioned effective dosages were able to regress the progression of cancerous cervix tissues [130].

### 2.11. Zerumbone and Pancreatic Cancer

Pancreatic carcinoma is a common cancer with a gradual increase in incidence rate [134]. Treatment of pancreatic cancer cells with zerumbone inhibited tube formation of human umbilical vein endothelial cells through the suppression of mRNA expression and proteins associated with angiogenic function and NF-κB activity [135]. Further, zerumbone reduced cell viability and induced apoptosis in PANC-1 cells, as evidenced the upregulated expression of p53, p21 protein, and elevated ROS levels [134]. In addition, zerumbone caused downregulation in the expression of CXCR4, which correlated with suppression of CXCL12-induced invasion [61].

### 2.12. Zerumbone and Prostate Cancer

Prostate cancer is the one of the most common causes of malignancy in males, and is without curative options in the advanced state [136,137,138,139,140,141,142,143]. Pre-treatment of prostate cancer cells with zerumbone significantly decreased the radiation-induced expression of phosphorylated ATM (ataxia telangiectasia-mutated) and suppressed the expression of JAK2 and STAT3, which are involved in DNA damage repair [144]. In addition, zerumbone selectively inhibited the IL-6/JAK2/STAT3 pathway and blocked the prostate cancer-associated genes- cyclin D1, IL-6, COX2 (cytochrome c oxidase), and ETS Variant 1 (ETV1); thereby inducing cytotoxicity through G0/G1 cell cycle arrest and causing apoptosis [145]. Zerumbone also attenuated microtubule assembly and induced endoplasmic reticulum (ER) stress and MMP-2 expression in prostate cancer cells and upregulated the expression of GRP-78 and C/EBP homologous protein (CHOP)/ growth arrest and DNA damage 153 (GADD153). An increase in intracellular Ca^2+^ levels potentially acted as a crosstalk marker between this ER stress and mitochondrial insult, which was associated with the formation of the active calpain I fragment. Zerumbone also induced apoptosis and autophagy through the caspase-dependent pathway and LC3-II formation [43]. 

### 2.13. Zerumbone and Renal Cell Carcinoma

Renal cell carcinoma (RCC) is a malignant disease insensitive to conventional treatments, contributing >90% of the most common form of kidney cancer [146,147]. Zerumbone showed its anti-cancer effects by initiating apoptosis through the activation of caspase-3 and caspase-9, leading to cleavage of PARP and downregulation of Gli-1 and Bcl-2 [146]. In addition, zerumbone could induce the expression of tyrosine phosphatase SHP-1(Src homology region 2 domain-containing phosphatase-1), which has been associated with inhibition of STAT3 activation thereby resulting in the suppression of the gene products that are involved with proliferation, survival, and angiogenesis. 

In line with the above, similar abrogation of STAT3 activation was exhibited in tumor growth and tissues upon administration of zerumbone in athymic nu/nu mice with an RCC xenograft. For this, the tumor tissues were treated with vehicle and 50 mg/kg body weight of zerumbone for 6-week duration and substantial inhibition of STAT3 activation was noted [148]. 

### 2.14. Zerumbone and Skin Cancer

Skin cancer is the most commonly diagnosed cancer that begins with an abnormal growth in the epidermal layer, and can be classified as melanoma and non-melanoma skin cancer [149]. Pre-treatment with zerumbone at the tumor promotion stage in mice suppressed tumor growth and the mechanism behind its effect might be due to increased expression of xenobiotic-metabolizing enzymes (GSTP1, NQO1) and mRNA levels for manganese superoxide dismutase (MnSOD) and glutathione peroxidase-1 (GPx1). In addition, zerumbone decreased the levels of cyclooxygenase-2 (COX-2) expression, ERK1 phosphorylation, H_2_O_2_-induced edema formation, and leukocyte infiltration [150]. 

Further, treatment of epidermal cells with zerumbone in mice enhanced the binding property of Nrf2 to the antioxidant element, which resulted in increased HO-1 activity, thus providing a basis for the reported antioxidant effects of this agent against skin carcinogenesis [63]. 

## 3. Cytotoxicity Data

The cytotoxicity data has been analyzed for both the in vitro and in vivo studies. The treatment of zerumbone on various cancers was reported to be effective in various in vitro studies. For the liver cancer, the IC_50_ of zerumbone in HepG2 cells was reported to be 6.20 µg/mL [116]. While the IC_50_ for cervical cancer in Hela cells was reported to be 6.4 µg/mL, and in breast cancer cell lines (MCF-7 and MDA-MB 231) 23.0 µg/mL and 24.3 µg/mL respectively [151]. In case of the in vivo studies, zerumbone suppressed the tumor growth and volume in different cancers with different dosage rates. In line with this, the treatment of zerumbone for a 6-week duration caused tumor growth inhibition in renal cancer model, the dosage was five times a week with 50 mg/kg body weight. The treatment also showed less substantial toxic effects as it did not cause weight loss in treated mice [148] while in cervical cancer models different groups were administered with 4 mg/kg, 8 mg/kg, and 16 mg/kg of zerumbone respectively. No remarkable regression of the CIN lesions was observed with 4 mg/kg dosage as compared to the later dosages. The dosage of 8 mg/kg showed antiproliferative properties while 16 mg/kg showed decreased in CIN lesions [130]. The dosage of zerumbone in breast cancer can also be compared where the mice models were treated with 20 mg/kg of zerumbone which resulted to smaller tumors than the control group treated with vehicle. The weight level of tumor in zerumbone treated mice was lesser with 1.22 ± 0.35 g as compared to the mice treated with vehicle that weighed 2.56 ± 0.68 g [77]. In case of colon and lung cancer studies, the mice models were injected with azoxymethane (10 mg/kg bw), promoted by 1.5% dextran sulphate sodium in drinking water for 7 days to initiate colon tumour while the other models were injected with 4-(methylnitrosamino)-1-(3-pyridyl)-1-butanone (10 micromol/mouse) to induce lung tumor. Both the tumor models were later administered with zerumbone at 100, 250, and 500 ppm for 17 weeks and 21 weeks, respectively, that resulted in inhibition of multiplicity the cancers [87].

## 4. Limitation and Future Prospects

Various potential studies of zerumbone against different tumor cell lines have been reported. However, most of the reports are mainly confined to in vitro studies. Few in vivo model studies are reported in only few cancers such as breast, colorectal, cervical, lung, renal cell carcinoma, and skin cancers. Also, relevant clinical trials to test the safety and efficacy of zerumbone are not available. The limitations in clinical studies might be due to few in vivo model findings. Further, the pharmacokinetic properties such as solubility, distribution, etc. of the compound should be analysed properly. A previous study that aimed to enhance the solubility of zerumbone was explored to analyse its interaction with hydroxypropyl-β-cyclodextrin (HPβCD) in aqueous media where it was showed that the solubility of zerumbone substantially increased with an augmentation in the concentration of HPβCD at 20 °C thereby suggesting that this complexation can be used in this drug formulation [50].

Therefore, apart from in vitro and in vivo studies, the pharmacokinetic properties of zerumbone can be investigated and established, and also clinical trial studies can be carried out further to validate its therapeutic application in cancer patients.

## 5. Conclusions

Despite the advancement in treatment methods, cancer is still one of the deadliest diseases causing havoc to human health. Most of the treatment methods available are less effective and often associated with severe side effects and emergence of chemoresistance. Therefore, it is essential to discover a new alternate, safe, and efficacious treatment method against cancer. Zerumbone, a monocyclic sesquiterpene, isolated from *Z. zerumbet* is a compound that has been reported for its diverse anti-cancer properties mediated by suppression of proliferation, induction of cell cycle arrest, and apoptosis in various cancers such as those of brain, breast, colon, liver, and lung through the modulation of various proteins and signalling pathways (Table 1). With regard to this, various in vitro studies have shown that zerumbone can downregulate the expression CXCR4, activation of NF-κB, and other oncogenic proteins. Additionally, zerumbone can also repress the IL-6/JAK2/STAT3, PI3K/AKT/mTOR pathways, and expression of associated genes such as *cyclin D1*, *IL-6*, *COX2*, and *ETV1*, thus inhibiting proliferation and angiogenic activity by increasing cell cycle arrest and apoptosis. Moreover in preclinical studies, administration of zerumbone in various mouse models was observed to tumor growth and metastasis. However, despite the availability of many pre-clinical studies on the anti-cancer properties of zerumbone, clinical trials with this compound have been rarely reported. Therefore, more preclinical studies would be required to establish clinical results and a better therapeutic potential of zerumbone, to target cancer. 

## Figures and Tables

**Table 1:** Possible role of zerumbone against various cancers.

Cancer	In Vitro/In Vivo	Model	Mechanism of Action	Reference
Breast cancer	In vitro	MDA-MB-231, MDA-MB-468, MDA-MB-361, T-47D	↓CD1d	[152]
	In vitro	MCF7, Hs578T, MDA-MB231	↓IL-1β	[72]
	In vitro	HCC1806	↓TGF-β1	[77]
	In vitro	SKBR3, MDA-MB468	↓CD44, ↓STAT-3	[71]
	In vitro	MCF-7, MDA-MB-231	↓Notch4	[79]
	In vitro	Hs578T, MDA-MB231	↓IL-8, ↓MMP-3	[80]
	In vitro	MDA-MB-231, MCF-7	↑Bax, ↑Bak	[60]
	In vivo	Mouse	↓Tumor growth	[60]
	In vitro	MDA-MB-231, U266	↓NF-κB	[64]
Cervical cancer	In vivo In vitro	Athymic mice HeLa, H9c2	↓Osteolysis ↑Caspase 3	[64] [8]
	In vivo	female BALB/c mice	↓Neoplasia	[133]
	In vivo	female BALB/c mice	↑apoptosis	[130]
Colon cancer	In vitro	Caco-2, Colo320DM, HT-29	↑IL-6	[85]
	In vitro	HCT116	↓TNF-α	[40]
	In vitro	HCT116	↑DR5, DR4, Caspase-8	[84]
	In vitro	HCT-116, SW-48	↓β-catenin	[153]
	In vitro	SW480	↑caspase 3, ↑caspase 8, ↑caspase 9	[3]
	In vitro	HCT116	↓GSH	[82]
	In vivo	Mice	↓multiplicity of adenomas	[87]
Gastric cancer	In vitro	SGC-7901	↓Bcl-2	[13]
	In vitro	MKN1, MKN28, MKN45, MKN74, NUGC4, AGS	↓NF-κB	[99]
Liver cancer	In vitro	HepG2	↑Bax	[117]
	In vitro	HepG2	↑p27, ↑Cyt-c, ↑caspase-3 & caspase-9	[154]
	In vitro	HepG2	↓VEGF, ↓MMP-9	[116]
	In vitro	HepG2, Hep3B, Sk-Hep-1, SNU-182, SNU-449	↓PI3K/AKT/mTOR, ↓STAT-3	[115]
	In vitro	HepG2	↑Cell cycle arrest	[155]
Leukemia	In vitro	CEM-ss	↑Caspase-3	[100]
Lung Cancer	In vitro	A549	↓FAK/AKT/ROCK	[38]
	In vitro	A549, NCI-H460	↑p53	[125]
	In vivo	Mice	↓Carcinogenesis	[87]
Oral cancer	In vitro	OSCC	↓PI3K-mTOR	[129]
Pancreatic cancer	In vitro	PaCa	↓NF-κB	[135]
	In vitro	PANC-1, SW1990	↑ROS	[134]
Prostate cancer	In vitro	PC3, DU145	↓Phosphorylated ATM	[144]
	In vitro	DU145, PC3	↓JAK2/STAT3	[145]
Renal cell carcinoma	In vitro	786-0,769-P	↓Gli-1/Bcl-2	[146]
	In vivo	Athymic nu/nu mice	↓STAT3, ↓Tumor growth	[148]
Skin cancer	In vitro	CHL-1	↑ROS	[156]
	In vitro	A375	↓Bcl-2	[59]

## Abbreviations

ATM	Ataxia-telangiectasia mutated
Bcl-2	B-cell lymphoma- 2
Bak	Bcl-2 homologous antagonist/killer
Bax	BCL2-associated X protein
CD1d	Cluster of differentiation 1
CD44	Cluster of differentiation 44
DR	Death receptor
Gli 1	Glioma-associated oncogene 1
GSH	Glutathione
IL-6	Interleukin 6
IL-8	Interleukin 8
IL-1β	Interleukin 1 beta
JAK2	Janus kinase 2
MMP-3	matrix metalloproteinase-3
MMP-9	Matrix metallopeptidase 9
mTOR	Mammalian target of rapamycin
NF-κB	nuclear factor kappa light chain enhancer of activated B cells
OSCC	Oral squamous cell carcinoma
ROCK	Rho-associated protein kinase
ROS	Reactive oxygen species
STAT-3	Signal transducer and activator of transcription 3
TGF-β1	Transforming growth factor beta 1
TNFα	Tumour necrosis factor alpha
VEGF	Vascular endothelial growth factor
